# Primary Tumor Resection Is Associated with Improved Survival in Stage IV Colorectal Cancer: An Instrumental Variable Analysis

**DOI:** 10.1038/srep16516

**Published:** 2015-11-13

**Authors:** Hong Xu, Zuguang Xia, Xiaoyan Jia, Kai Chen, Dapeng Li, Yun Dai, Min Tao, Yixiang Mao

**Affiliations:** 1 Department of Oncology, The First Affiliated Hospital of Soochow University, Suzhou, China; 2 Department of Gastrointestinal Medical Oncology, The University of Texas MD Anderson Cancer Center, Houston, Texas, USA; 3 Department of Medical Oncology, Fudan University Shanghai Cancer Center, Department of Oncology, Shanghai Medical College, Fudan University, Shanghai, China

## Abstract

Primary tumor resection (PTR) is recommended for patients with unresectable stage IV colorectal cancer (CRC) who present with symptoms related to their primary tumor. However, the survival benefit of PTR for asymptomatic patients is controversial. We investigated the change in PTR rates and the contribution of PTR to survival in patients with unresectable stage IV CRC over the past two decades in the United States. Clinicopathological factors and long-term survival were compared for 44 514 patients diagnosed with unresectable stage IV CRC from January 1, 1988, through December 31, 2010, who had or had not undergone PTR. Multivariable Cox regression and the instrumental variable method were used to identify independent factors for survival. Of the 44 514 patients with unresectable stage IV CRC, 27 931 (62.7%) had undergone PTR. The annual rate of PTR decreased from 74.4% to 50.2% diagnosed in 1988 and 2010, and the median overall survival increased for both PTR and non-PTR patients. Instrumental variable analyses revealed that PTR was associated with better overall, cancer-specific, and other-cause survival of patients with unresectable stage IV CRC.

Colorectal cancer (CRC) is the fourth most common type of cancer in the United States, with an estimated incidence of 136 830 new cases and 50 310 deaths in 2014^1^. Due to cancer screening efforts and improvements in chemotherapy, both the incidence and the death rate of CRC have decreased in the past 10 years^2^. Approximately 20% of CRC patients, however, present with synchronous metastases^3^. Once CRC has metastasized to another part of the body, the chances of curing it dramatically decline. The 5-year relative survival for metastatic CRC is 12.9%^1^.

Patients with a resectable primary colorectal tumor and resectable synchronous metastases can be treated with staged or simultaneous resection. However, in patients presenting with unresectable metastases and with few or absent symptoms, the indication for prophylactic resection is under debate, and the effectsof such resection on survival and quality of life are uncertain^4^,^5^,^6^,^7^. The CRC panel of the National Comprehensive Cancer Network believes that the risks of surgery outweigh the possible benefits of resection of asymptomatic primary tumors, and routine palliative resection of a synchronous primary lesion should therefore be considered only if the patient has an unequivocal imminent risk of obstruction or acute significant bleeding. Systemic chemotherapy is the preferred initial strategy, and patients on regimens deemed to be potentially convertible should be reevaluated for resection every 2 months^8^. Furthermore, complications from an intact primary lesion are uncommon^9^, and removal of the lesion delays initiation of systemic chemotherapy.

Prolongation of overall survival in patients with unresectable stage IV CRC may increase the patients’ exposure to possible complications from the primary tumor. Hence, there may be a potential advantage in removing the asymptomatic primary tumor in such patients. The results from a randomized controlled trial would help answer the question of whether and/or when primary tumor resection (PTR) is indicated in patients with stage IV CRC. However, two recent randomized controlled trials on this topic (NCT01086618 and ACTRN12609000680268) were canceled because of insufficient recruitment of patients due to aversion to randomization. We therefore performed a retrospective study to determine the PTR rate in patients with stage IV CRC in the United States and to evaluate the effect of PTR on survival in such patients.

## Materials and Methods

### Data Source

We abstracted data from the National Cancer Institute’s Surveillance, Epidemiology, and End Results (SEER) 18 registries database (November 2013 submission)^10^. In total, the database covers approximately 27.8% of the US population (based on the 2010 Census).Different years of diagnosis are included for different registries, ranging from 1973 to 2011. The data reported in this study represent the most recent follow-up (December 31, 2011) available in the SEER database.

### Cohort

We used SEER*Stat (version 8.1.5) to generate a case list. Patients aged 18–90 years with histologically confirmed first primary stage IV colorectal adenocarcinoma diagnosed between January 1, 1988, and December 31, 2010, were eligible to be included in the study. We used code 4 (distant) in SEER historic stage A to identify patients who had presented with metastatic disease. This SEER submission includes cases through December 31, 2011, which would represent either the date of the last cancer diagnosis or the date of last follow-up. However, we excluded patients diagnosed in 2011 because those patients had less than 1 year of potential follow-up time available (as the data cutoff date is December 31, 2011).The exclusion criteria were survival time of less than 30 days after a confirmed diagnosis; CRC being not the first diagnosis of malignant disease; and cancer reported from a nursing home, hospice, autopsy, or death certificate. To minimize the number of patients who had received surgery with curative intent, we also excluded patients who had undergone partial or total removal of other organs during PTR.

We generated a case list with information on the following variables: year of diagnosis, age at diagnosis, sex, race/ethnicity, marital status at diagnosis, tumor grade, site recode ICD-O-3/WHO 2008, vital status recode (study cutoff used), survival months, survival months flag (see below), reason no cancer-directed surgery, SEER cause-specific death classification, and SEER other-cause-of-death classification. The last two variables indicate whether the person died of the cancer (cause-specific survival) or causes other than the cancer. Because patients cannot be identified from the data, the Institutional Review Board of the First Affiliated Hospital of Soochow University exempted this study from review.

### Treatment Variables

PTR was defined as partial, subtotal, or total colectomy; proctectomy; or total proctocolectomy without partial or total removal of other organs. Non-PTR patients were those who had had no surgery, local tumor destruction, or excision^11^.

### Outcome Measures

We used the vital status recode (study cutoff used), SEER cause-specific death classification, and SEER other-cause-of-death classification variables to extract data on the status of patients at the time of last follow-up. Based on this information, we calculated overall, cause-specific, and other-cause survival rates. We used the survival months variable to extract information on time from the date of diagnosis to last follow-up. SEER*Stat estimates survival time in months by subtracting the date of diagnosis from the date of death or last contact. We used the survival months flag variable to identify missing or incomplete survival data. We excluded patients who had conflicting data or who died prior to recommended surgery.

### Statistical Analysis

Clinicopathologic factors of patients with stage IV CRC who had and had not undergone PTR were compared by χ^2^ tests. The PTR rate was calculated for each year in the study period. Survival times for different strata were compared by using the log-rank test. We created separate Kaplan-Meier survival curves for PTR and non-PTR patients. The significance of trends in values was determined with the Cochran–Armitage trend test. We performed a univariate Cox proportional hazards regression analysis to determine the hazard ratios (HRs) of death with respect to year of diagnosis, age, sex, race/ethnicity, marital status, tumor grade, tumor location, and PTR. We performed multivariable analyses on the association between PTR and survival duration, adjusting for all the applicable confounders listed above.

We used a two-stage residual inclusion estimation framework for instrumental-variable analysis to adjust for unmeasured confounding^12^,^13^. Health Service Area (HSA) definitions were linked to the SEER data by county of residence, allowing the patients to be organized into contiguous clusters of counties that correspond to geographic markets for health care. We used the National Cancer Institute modification, which defines HSAs within specific SEER catchment areas^14^. We created a five-category instrumental variable by grouping patients into quintiles on the basis of the rate of PTR use in the patient’s HSA, with quintile 1 corresponding to the lowest rate of PTR, and quintile 5 corresponding to the highest rate of PTR. We excluded patients who lived in HSAs with fewer than 10 patients, because we could not be certain that the PTR rates for those HSAs were accurate. HSA-level utilization has been used previously as an effective instrumental variable in cases where the use of an intervention of interest varied by hospital^15^,^16^.

To assess the validity of PTR use per HSA as an instrumental variable, we confirmed that percent PTR use in an HSA was highly correlated with likelihood of a PTR-eligible patient in that HSA having undergone PTR (F statistic > 10)^17^ but not associated with survival in a standard multivariable proportional hazards model. We also examined covariate balance across quintiles. In the first-stage model, we measured the association between PTR and the instrumental variable, adjusting for patient-level covariates, including surgery. From this model, we determined the raw residual for each patient by calculating the difference between the model-predicted probability of undergoing PTR and the actual treatment received. The residuals were then included as an additional covariate in our second-stage survival model.

All *P* values were two-tailed. A *P* value less than 0.05 was considered statistically significant. All statistical analyses were performed using SPSS version 22.0 (IBM Inc., Armonk, NY).

## Results

### Patient Characteristics

**Figure 1:**
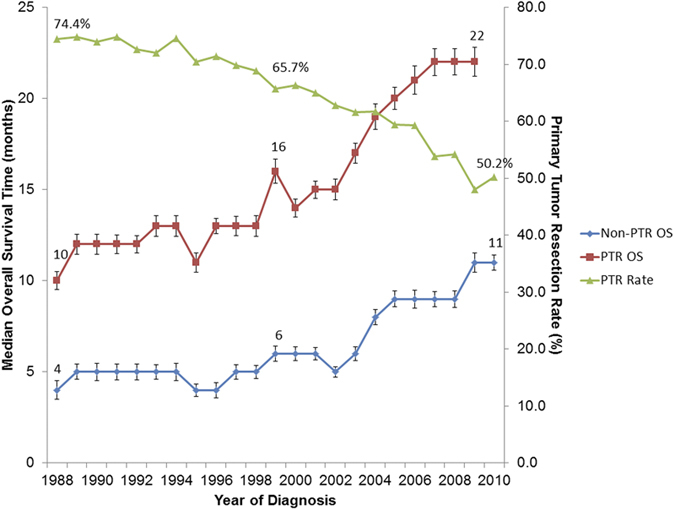
Primary Tumor Resection Rates and Median Overall Survival Times for Patients with Stage IV Colorectal Cancer. Abbreviations: OS, overall survival; PTR, primary tumor resection. *P*_trend_ values for median overall survival times of PTR and non-PTR patients relative to the year of diagnosis were less than 0.001. The median overall survival time of PTR patients with CRC diagnosed in the year 2010 was not met.

We identified 50  879 patients with first primary stage IV CRC diagnosed between 1988 and 2010.We excluded 6  262 patients with inconsistencies in the form of a record of Surgery of othreg/dis sites (1998–2002) or RX Summ–SurgOthReg/Dis (2003+) and RX Summ–Surg Prim Site (1998+) variables, diagnosed later than 1998 with a record of surgery at non-primary sites. Thirty-five patients who died prior to recommended surgery were also excluded, as were 68 patients who lived in 15 HSAs with fewer than 10 patients eligible for PTR. In summary, 44 514 patients in 187 HSAs were included. PTR rates ranged from 39.5% to 84.6% across the 187 HSAs (median 62.5%). The median age at diagnosis was 66 years (interquartile range 56–75 years). The overall median follow-up time was 85 months. Overall, 62.7% of the patients had undergone PTR. Compared with patients who had not undergone PTR, those who had undergone PTR were more likely be white, married or to have the tumor in the colon. The PTR rates for patients with stage IV CRC diagnosed in 1988 or 2010 were 74.4% and 50.2%, respectively (Fig. 1), representing a significant decrease in the use of PTR over time (*P*_trend_ < 0.001).

### Survival Analysis

#### Standard Cox Proportional Hazards Models

**Figure 2:**
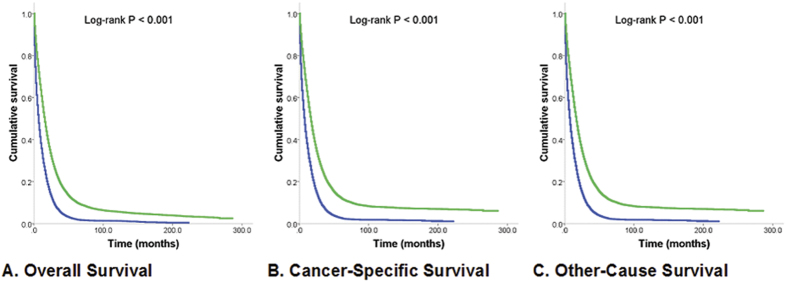
Kaplan-Meier Estimates of All-Cause, Cancer–Specific, and Other-Cause Mortality for Patients Who Had Undergone Primary Tumor Resection and Patients Who Had Not. Median survival times were compared using the log-rank test. **(A)** Overall survival. Green line, PTR patients; 24 570 deaths/27 931 patients; 16 months. Blue line, non-PTR patients; 15 494 deaths/16 583 patients; 7 months. **(B)** Cancer-specific survival. Green line, PTR patients; 22 728 deaths/27 931 patients; 17 months. Blue line, non-PTR patients; 14 572 deaths/16 583 patients; 8 months. **(C)** Other-cause survival. Green line, PTR patients; 1 842 deaths/27 931 patients; 229 months. Blue line, non-PTR patients; 922 deaths/16 583 patients; 161 months.

The median overall survival time for stage IV CRC patients who had PTR improved from 10 months to 22 months for patients diagnosed in 1988 and 2009 (*P* < 0.001). Survival for 2010 was not reported owing to limited follow-up time. Overall survival for non-PTR patients also increased from 4 to 11 months in the same period (*P* < 0.001) (Table 1 and Fig. 1). The median overall survival time for the entire cohort of patients with stage IV CRC was 12 months (95% confidence interval [CI]11.8–12.2). The median overall survival times for patients who had undergone PTR and patients who had not undergone PTR were 16 months (95% CI, 15.7–16.3) and 7 months (95% CI, 6.8–7.2), respectively (Fig. 2). *P*_trend_ values for median overall survival times of PTR and non-PTR patients with respect to the year of diagnosis were less than 0.001. In a multivariable analysis with adjustment for year of diagnosis, age, sex, race/ethnicity, marital status, tumor grade, and tumor location, the risk for all-cause death was higher for patients who had not undergone PTR than for those who had (HR 0.45, 95% CI 0.44–0.46). Furthermore, patients with PTR had longer cancer-specific (HR 0.44, 95% CI 0.43–0.45) and other-cause survival (HR 0.57, 95% CI 0.52–0.62) (Table 2). The following characteristics were associated with longer survival: young age; Asian ethnicity; well or moderate tumor differentiation; tumor location in the rectosigmoid, rectum, or left colon; and recency of diagnosis (Table 1). Male patients had longer overall survival in univariate analysis (HR 0.95, 95% CI 0.93–0.97); however, this effect was reversed after adjustment with other confounders. In standard Cox regression analyses, male patients seemed to have shorter overall (HR 1.02, 95% CI 1.00–1.04) and other-cause median survival times (HR 1.23, 95% CI 1.13–1.33) but not a shorter cancer-specific median survival time (HR 1.01, 95% CI 0.99–1.03).

#### Instrumental Variable Analysis

Patients were divided into quintiles based on the percentage of patients within each HSA who had undergone PTR. The average HSA PTR rate ranged from 51.4% (quintile 1) to 73.7% (quintile 5). Clinicopathological variables across quintiles are listed in Table 3. The instrumental variable was strongly associated with the use of PTR (χ^2^ test, *P* < 0.001). This relationship persisted in a multivariable model adjusted for all measured patient characteristics (F statistic = 566.04). Furthermore, in a standard proportional hazards survival model, we observed no independent association between the instrumental variable and overall survival (HR 0.99, 95% CI 0.98–1.00, *P* = 0.09). Taken together, these findings suggest that HSA PTR rate satisfies the two principal requirements for a valid instrument.

The survival benefit observed for PTR remained in the two-stage residual inclusion model. Estimates based on this instrument indicate that patients treated with PTR had a significantly lower likelihood of all-cause death than those treated without PTR (HR 0.50, 95% CI 0.49–0.52). We also found significant differences in cancer–specific (HR 0.50, 95% CI 0.49–0.51) and other-cause survival times (HR 0.54, 95% CI 0.49–0.59) between treatment groups (Table 2).

## Discussion

Our population-based study demonstrated a trend toward a lower PTR rate in patients presenting with unresectable stage IV CRC as well as better survival of patients with or without PTR over time. Survival analysis showed that being younger; being Asian; having well or moderately differentiated tumors; having the tumor in the rectosigmoid, rectum, or left colon; being recently diagnosed; or having undergone PTR was associated with longer overall survival. After adjustment for confounders, PTR was an independent factor of good prognosis for all-cause, cancer-specific, and other-cause survival in both standard Cox regression and instrumental variable models.

Currently, the recommended initial treatment for unresectable stage IV CRC is systemic chemotherapy^8^. The survival benefit of PTR in asymptomatic patients with unresectable stage IV CRC is controversial. A recent study by Hu *et al.* showed that the relative survival rate of patients presenting with stage IV CRC improved over time, as the PTR rate decreased^11^. Those authors therefore suggested that PTR may be overused. However, they did not perform a primary comparative analysis of the effect of PTR on survival and argued that such analysis with SEER data is subject to considerable selection bias that cannot be mitigated using standard multivariable regression techniques and that would lead to potential for misinterpretation. We conducted a head-to-head analysis using the SEER data in the same period and introduced percent PTR per HSA as an instrumental variable to reduce the interference of potential confounders. Our results did not agree with those reported by Hu *et al.* and revealed that PTR was an independent good prognostic factor for all-cause, cancer-specific, and other-cause survival in patients with stage IV CRC, despite the decrease in the rate of noncurative PTR over the last 2 decades.

Our study had several limitations. First, although our goal was to include only data for a first primary CRC, we found that other-site cancer deaths were included in the variable cancer-specific cancer survival. It seems that cancer-associated deaths may not be correctly reported in SEER. Second, although we had rich data on patient and tumor characteristics, we lacked data on potential confounders, such as performance status and chemotherapy. Nevertheless, to the extent that patients in different HSAs have generally similar performance statuses and other characteristics, the lack of such information was not likely to influence our results. Finally, SEER provides no information regarding the reasons for PTR, while providing reasons for decisions not to perform cancer-directed surgery. Patients in our study who had undergone PTR could have undergone palliative resection or resection of metastasis with curative intent. However, the relationship between PTR and longer survival was not undermined when we used the HSA PTR rate as an instrumental variable to mimic randomization to therapy.

There are several potential explanations for the observed association between increased survival time and PTR in patients with stage IV CRC. First, the improvement in survival following PTR may be attributed to a better response to chemotherapy after reduction of tumor burden^18^. This has been demonstrated by the proven benefit of resecting primary renal and ovarian tumors in the presence of metastatic disease^19^,^20^. Survival of resection patients may also improve because the patients are less likely to develop obstruction and perforation, complications known to be associated with high operative mortality and morbidity^21^. Moreover, surgical removal of the primary tumor may restore immunocompetence, even in patients with metastatic disease^22^. Patients with unresectable stage IV CRC may also survive longer after PTR due to changes in clinicopathological factors such as reduced comorbidities, improved performance status, and reduced nutritional deficiencies. However, we did not observe differences in available clinicopathological factors between PTR patients and non-PTR patients in our analyses.

We have demonstrated that in patients with stage IV CRC, the use of PTR decreased and survival increased between 1988 and 2010. Subgroup analysis showed that the survival times improved over time in both PTR and non-PTR patients. The benefit of PTR remained with the addition of more effective chemotherapeutic options. Our findings revealed that PTR may improve not only overall survival but also cancer-specific and other-cause survival of patients with stage IV CRC. Prospective trials are needed to confirm the effectiveness of PTR in patients with stage IV CRC, especially in asymptomatic patients.

## Additional Information

**How to cite this article**: Xu, H. *et al.* Primary Tumor Resection Is Associated with Improved Survival in Stage IV Colorectal Cancer: An Instrumental Variable Analysis. *Sci. Rep.* **5**, 16516; doi: 10.1038/srep16516 (2015).

## Figures and Tables

**Table 1:** Correlation between Clinicopathological Factors and Median Survival Time in Patients with Stage IV Colorectal Cancer Who Had Not Undergone Nonprimary-Tumor Surgery.

Characteristic	No. of Patients	No. of Deaths	MST (months)	Log-rank	HR (95% CI)
Age at diagnosis, y, No.				<0.001	
18–49	6 055	5 050	17		1 [Reference]
50–75	27 756	24 788	13		1.22 (1.18–1.25)
76–90	10 703	10 226	7		1.86 (1.80–1.93)
Sex, No.				<0.001	
Female	19 258	17 328	11		1 [Reference]
Male	25 256	22 736	13		0.95 (0.93–0.97)
Race/ethnicity, No.				0.019	
White	34 623	31 239	12		1 [Reference]
Black	5 887	5 377	11		1.11 (1.08–1.14)
Asian	3 329	2 863	14		0.89 (0.86–0.93)
Others and unknown	675	585	13		0.94 (0.87–1.02)
Marital status, No.				<0.001	
Single	6 707	5 985	11		1 [Reference]
Married	24 361	21 724	14		0.90 (0.87–0.92)
Separated, divorced, or widowed	12 039	11 148	9		1.13 (1.09–1.16)
Unknown	1 407	1 207	12		1.00 (0.94–1.06)
Tumor grade, No.				0.017	
Poorly differentiated or undifferentiated	10 639	9 850	9		1 [Reference]
Well or moderately differentiated	26 885	23 709	15		0.73 (0.71–0.75)
Unknown	6 990	6 505	7		1.17 (1.14–1.21)
Tumor location, No.				0.046	
Right colon, including transverse colon	10 745	9 854	9		1 [Reference]
Left colon	16 165	14 334	14		0.77 (0.75–0.79)
Rectosigmoid or rectum	14 990	13 424	13		0.81 (0.79–0.84)
Not otherwise specified	2 614	2 452	5		1.35 (1.29–1.41)
Year of diagnosis, No.				<0.001	
1988–1991	4 208	4 148	9		1 [Reference]
1992–1994	4 001	3 915	10		0.97 (0.93–1.01)
1995–1997	4 012	3 919	9		0.99 (0.95–1.03)
1998–2000	5 046	4 876	10		0.92 (0.88–0.96)
2001–2003	8 074	7 710	11		0.86 (0.83–0.89)
2004–2006	8 014	7 366	15		0.72 (0.69–0.75)
2007–2010	11 159	8 130	16		0.67 (0.65–0.70)
Primary tumor resection				<0.001	
No	16 583	15 494	7		1 [Reference]
Yes	27 931	24 570	16		0.54 (0.53–0.55)

Abbreviations: CI, confidence interval; HR, hazard ratio; MST, median survival time. Survival was analyzed by using Kaplan-Meier curves and the univariate Cox regression test.

**Table 2:** Multivariable Analyses of Survival in Patients with Stage IV Colorectal Cancer Who Had or Had Not Undergone Primary Tumor Resection.

	PTR rate (%)	HR (95% CI) in PTR patients compared with non-PTR patients					
		Standard Cox regression analysis			Instrumental variable analysis^a^		
		Overall survival	Cancer-specific survival	Other-cause survival	Overall survival	Cancer-specific survival	Other-cause survival
Primary analysis							
All patients	27 931/44 514 (62.7)	0.45 (0.44–0.46)	0.44 (0.43–0.45)	0.57 (0.52–0.62)	0.50 (0.49–0.52)	0.50 (0.49–0.51)	0.54 (0.49–0.59)
Subgroup analyses							
Patients aged ≤ 65 y	13 902/22 058 (63.0)	0.44 (0.43–0.45)	0.44 (0.42–0.45)	0.52 (0.45–0.61)	0.51 (0.49–0.53)	0.50 (0.48–0.52)	0.64 (0.55–0.75)
Patients aged > 65 y	14 029/22 456 (62.5)	0.46 (0.44–0.47)	0.45 (0.43–0.46)	0.57 (0.51–0.64)	0.49 (0.47–0.51)	0.49 (0.48–0.51)	0.47 (0.42–0.53)
Sensitivity analyses							
Metro counties	10 587/14 744 (71.8)	0.45 (0.44–0.46)	0.44 (0.43–0.45)	0.56 (0.50–0.61)	0.50 (0.49–0.51)	0.49 (0.48–0.51)	0.54 (0.49–0.60)
Nonmetro counties	17 344/29 770 (58.3)	0.47 (0.44–0.50)	0.46 (0.43–0.49)	0.61 (0.47–0.80)	0.52 (0.49–0.56)	0.54 (0.50–0.57)	0.48 (0.36–0.64)
Diagnosis years 1988–1999	23 758/38 013 (62.5)	0.44 (0.42–0.45)	0.43 (0.41–0.45)	0.50 (0.43–0.59)	0.46 (0.44–0.48)	0.46 (0.44–0.48)	0.42 (0.36–0.49)
Diagnosis years 2000–2010	4 173/6 501 (64.2)	0.47 (0.46–0.49)	0.46 (0.45–0.48)	0.61 (0.54–0.68)	0.55 (0.53–0.56)	0.55 (0.53–0.56)	0.57 (0.51–0.64)

Abbreviations: HR, hazard ratio; PTR, primary tumor resection.

^a^HRs were derived from a two-stage residual inclusion model using the Weibull distribution. Multivariable survival analyses were adjusted for year of diagnosis, age, sex, race/ethnicity, marital status, tumor grade, and tumor location when applicable.

**Table 3:** Characteristics of Patients Relative to Health Services Area Rates of Primary Tumor Resection.

	HSA PTR Rate Quintile					*P*^a^
	1	2	3	4	5	
No. of patients	3 804	7 586	17 618	12 671	2 835	
PTR rate (average %)	51.4	58.0	63.1	66.1	73.7	
Median survival (months)	13	13	12	12	12	
Age at diagnosis, y (%)						
18–49	13.9	14.8	13.3	13.1	14.2	0.008
50–75	61.1	62.4	62.4	62.6	62.3	
76–90	25.0	22.8	24.3	24.2	23.5	
Sex (%)						
Female	43.1	42.3	43.8	43.1	43.6	0.301
Male	56.9	57.7	56.2	56.9	56.4	
Race/ethnicity (%)						
White	79.0	78.5	76.9	75.8	88.4	<0.001
Black	16.5	10.1	14.9	12.4	10.4	
Asian	3.2	7.8	7.5	10.1	0.9	
Others and unknown	1.3	3.6	0.7	1.8	0.2	
Marital status (%)						
Single	15.6	15.5	15.7	14.4	12.4	<0.001
Married	53.7	53.2	54.1	55.8	59.4	
Separated, divorced, or widowed	26.4	27.5	27.3	26.8	26.5	
Unknown	4.3	3.9	3.0	3.0	1.7	
Tumor grade (%)						
Poorly differentiated or undifferentiated	22.5	21.5	23.9	24.8	27.9	<0.001
Well or moderately differentiated	59.3	62.2	60.2	59.7	61.2	
Unknown	18.3	16.2	15.9	15.4	10.9	
Tumor location (%)						
Right colon, including transverse colon	22.0	23.3	24.1	25.3	24.6	<0.001
Left colon	33.9	35.8	36.7	36.8	36.2	
Rectosigmoid or rectum	36.0	35.0	33.5	32.4	34.2	
Not otherwise specified	8.1	5.9	5.7	5.6	5.0	
Year of diagnosis (%)						
1988–1991	1.0	2.3	8.6	16.3	14.7	<0.001
1992–1994	0.7	2.0	11.6	11.8	9.9	
1995–1997	0.9	2.1	11.3	11.9	11.6	
1998–2000	8.7	8.7	12.6	12.3	10.0	
2001–2003	28.0	24.5	16.8	13.8	15.7	
2004–2006	24.6	25.6	16.6	14.0	15.6	
2007–2010	36.1	34.8	22.7	19.8	22.5	

Abbreviations: HSA, health service area; PTR, primary tumor resection.

^a^χ^2^ test of independence.
